# Protein and Amino Acid Adequacy and Food Consumption by Processing Level in Vegans in Brazil

**DOI:** 10.1001/jamanetworkopen.2024.18226

**Published:** 2024-06-24

**Authors:** Alice Erwig Leitão, Gabriel P. Esteves, Bruna Caruso Mazzolani, Fabiana Infante Smaira, Martin Hindermann Santini, Heloísa C. Santo André, Bruno Gualano, Hamilton Roschel

**Affiliations:** 1 Applied Physiology and Nutrition Research Group–School of Physical Education and Sport and Faculdade de Medicina, Universidade de Sao Paulo, São Paulo, São Paulo, Brazil; 2 Center of Lifestyle Medicine; Laboratory of Assessment and Conditioning in Rheumatology, Hospital das Clínicas, Faculdade de Medicina, Universidade de Sao Paulo, São Paulo, São Paulo, Brazil; 3 Food Research Center, University of São Paulo, São Paulo, Brazil; 4 School of Applied Sciences, State University of Campinas, Limeira, Brazil

## Abstract

**Importance:**

Major concerns regarding individuals who adhere to a vegan diet are whether they meet protein and essential amino acid recommendations and how reliant they are on ultraprocessed foods.

**Objectives:**

To investigate whether individuals who adhere to a vegan diet meet protein and essential amino acid recommendations and, as secondary objectives, to determine ultraprocessed food intake and potential factors associated with inadequate protein intake in this population.

**Design, Setting, and Participants:**

This cross-sectional survey study was conducted between September 2021 and January 2023 in Brazil among male and female adults (aged 18 years or older) who adhered to a vegan diet recruited from social media platforms.

**Exposure:**

Adherence to a vegan diet and unprocessed and minimally processed foods and ultraprocessed food consumption.

**Main Outcomes and Measures:**

Protein and essential amino acid intake and food consumption by processing level were assessed using a 1-day food diary. Nutrient adequacy ratios were calculated by dividing nutrient intake by its recommendation (using scores truncated at 1) for each participant and then finding the mean across participants for each nutrient. The mean adequacy ratio was the mean of all nutrient adequacy ratios.

**Results:**

Of 1014 participants who completed the survey, 774 individuals (median [IQR] age, 29 [24-35] years; 637 female [82.3%]) were confirmed as adhering to a vegan diet and provided adequate food recalls, among whom 558 individuals reported body weight and so had relative protein and amino acid intake values available. The median (IQR) body mass index (calculated as weight in kilograms divided by height in meters squared) of participants was 22.6 (20.3-24.8). The nutrient adequacy ratio of protein was 0.93 (95% CI, 0.91-0.94); for essential amino acids, ratios ranged from 0.90 (95% CI, 0.89-0.92) for lysine to 0.98 (95% CI, 0.97-0.99) for phenylalanine and tyrosine. The mean adequacy ratio for protein and all amino acids was 0.95 (95% CI, 0.94-0.96). The median intake level was 66.5% (95% CI, 65.0%-67.9%) of total energy intake for unprocessed and minimally processed food and 13.2% (95% CI, 12.4%-14.4%) of total energy intake for ultraprocessed food. Adjusted logistic regression models showed that consuming protein supplements (odds ratio [OR], 0.06 [95% CI 0.02-0.14]; *P* < .001) or textured soy protein (OR, 0.32 [95% CI, 0.17-0.59]; *P* < .001) was associated with decreased odds of inadequate protein intake. Higher ultraprocessed food intake levels were also associated with decreased odds of inadequate protein intake (eg, fourth vs first quartile of intake: OR, 0.16 [95% CI, 0.07-0.33]; *P* < .001), and higher unprocessed and minimally processed protein intake levels were associated with increased odds of inadequate protein intake (eg, fourth vs first quartile of intake: OR, 12.42 [95% CI, 5.56-29.51]; *P* < .001).

**Conclusions and Relevance:**

In this study, most individuals who adhered to a vegan diet attained protein and essential amino acid intake recommendations, largely based their diet of unprocessed and minimally processed food, and had a significantly lower proportion of ultraprocessed food intake compared with previous reports. Participants consuming less ultraprocessed food were more likely to have inadequate protein intake, suggesting a significant reliance on ultraprocessed proteins for this population.

## Key Points

**Question:**

Do individuals who adhere to a vegan diet meet protein and essential amino acid needs, and are they reliant on ultraprocessed foods?

**Findings:**

In this cross-sectional survey study of responses to a 1-day food diary from 774 adults who adhered to a vegan diet, including 558 participants with body weight data, the mean protein and amino acid adequacy ratio comparing nutrient intake vs recommendations was 0.95; unprocessed or minimally processed and ultraprocessed foods accounted for 66.5% and 13.2% of energy intake, respectively. Protein supplements, textured soy protein, and ultraprocessed food were associated with decreased odds of protein inadequacy and unprocessed and minimally processed protein with increased odds of protein inadequacy.

**Meaning:**

This study found that most individuals who adhered to a vegan diet attained protein recommendations but were less likely to do so when consuming less ultraprocessed food.

## Teaser

This cross-sectional survey study compares protein and essential amino acid intake levels against recommended levels among individuals in Brazil who adhere to a vegan diet and assesses whether adequate protein consumption is associated with greater intake of processed foods.

## Introduction

Veganism is a lifestyle increasing in popularity worldwide that supports abstaining from the use of animal products. This leads to a dietary pattern that excludes meat, fish, poultry, dairy, eggs, and honey, among other animal-derived foods (henceforth, *vegan diet*).^1,2^

Refraining from protein-rich animal foods has generated ongoing controversy as to whether individuals who adhere to a vegan diet can adequately meet protein requirements.^3^ Although studies show that these individuals can have borderline-adequate protein intake, albeit slightly reduced vs individuals consuming omnivorous diets,^4^ it is unknown whether protein sources habitually consumed by vegans allow for adequate essential amino acid intake.

A range of plant-based meat and dairy substitutes have been formulated with the claim of being practical, protein-rich complements to a vegan diet. However, overconsumption of these products may disrupt the alimentary basis of vegan diets (unprocessed and minimally processed plant-based foods, such as fruits, vegetables, and whole grains) and lead to an increase in ultraprocessed food intake.^5,6^ The association of ultraprocessed food intake with overall health has been widely debated,^7,8,9^ warranting the investigation of food consumption as a function of processing level in the vegan population. Therefore, the primary aim of this study was to describe protein and essential amino acid intake in a survey of Brazilian individuals who follow a vegan diet. The secondary aim was assessing food intake by processing level according to the Nova classification system and investigating potential factors associated with protein inadequacy.

## Methods

This cross-sectional survey study was approved by the Centro Universitário Estácio de Sá de Santa Catarina ethical committee and conducted according to the Helsinki declaration. All participants signed a digital informed consent form. This manuscript adheres to the Strengthening the Reporting of Observational Studies in Epidemiology—Nutritional Epidemiology (STROBE-nut) reporting guideline and American Association for Public Opinion Research (AAPOR) reporting guideline for surveys.^10^

### Study Design and Participants

Data in this study derive from the Vegan Eating Habits and Nutritional Evaluation Survey (VEGAN-EATS), a cross-sectional survey conducted between September 2021 and January 2023. We previously published an analysis of disordered eating attitudes and food choice motives in this population stemming from this survey.^11^ This study expands on the topic by investigating the dietary profile of Brazilian vegan diets, with the topical issue of protein, essential amino acid intake, and food processing in this population.

Participants were recruited through advertisements on social media platforms and included males and females aged 18 years or older currently living in Brazil with internet access and the ability to read. Participants completed an online survey using the Google Forms platform (Google LLC).

### Evaluation Tool

The survey included questions regarding participant general characteristics, self-reported anthropometric data, and vegan lifestyle. General characteristics included educational level (elementary school, high school, college, or postgraduate education), income (categorized according to the Brazilian Institute of Geography and Statistics in levels of A [≥US $4200], B [US $1350-$4200], C [US $550-$1350], and D or E [≤US $550]),^12^ smoking (yes or no) and drinking status (none, 1-2 times/mo, 2-4 times/mo, 2-3 times/wk, or ≥ 4 times/wk), and exercise habits (does not exercise, 1-2 h/wk, 3-4 h/wk, 5-6 h/wk, or ≥7 h/wk). Vegan lifestyle–related questions were the duration of adherence to a vegan diet (< 1 y, 1-2 y, 2-3 y, 3-4 years, and >5 y) and main motivation to shift toward a vegan diet (ethics, health reasons, environment, politics, life philosophy, medical restrictions, religion, or sports performance). Macronutrient and micronutrient, amino acid, and food intake by processing level were assessed by a self-administered 1-day food diary. This tool has been previously shown to perform adequately compared with the typical three 24-hour recalls,^13^ and it was selected due to the impossibility of implementing repeated 24-hour recalls within our study. Participants received an instructional video on how to fulfil the diary and fully report quantity and type of foods and beverages consumed within the previous 24 hours, including a precise level of detail on food preparation and ingredients, allowing best practices for Nova food classification.^14,15^ Total energy (kilocalories), macronutrient amounts (grams), and relative contributions of each Nova food processing category to the total energy intake were calculated. We also calculated the relative contribution stemming from proteins by food processing category. Examples of commonly reported foods and their classification are available in eTable 1 in Supplement 1.

Food diaries were quantified with Nutritionist Pro software version 7.3 (Axxya Systems) using the US Department of Agriculture database. When nutritional information was not available, we searched the literature or directly contacted food companies. Amino acid composition was available for most consumed proteins (94.2%).

### Statistical Analysis

Due to missingness in body weight and height (available in 558 individuals), individuals with and without these data were compared and analyzed for potential associations between missingness and variables (eTable 2 in Supplement 1). There were only subtle differences between subgroups across all variables. Given that protein and amino acid intake adequacy requires assessing intake relative to body weight, observations with missing variables were excluded and complete cases were used for the main analysis. We also provided a complementary analysis in which missing body weight was imputed through multiple imputation^16^ using the mice package^17^ in R statistical software set to 5 iterations and the classification and regression trees method. A matrix of factors associated with body weight within the dataset was selected (age, sex, income class, exercise habits, motivation to shift to a vegan diet, total energy intake, and protein intake). The mean body weight from these 5 imputed datasets was used for descriptive statistics and model adjustment.

Descriptive data are presented as median and IQR for continuous variables and absolute and relative frequency (number and percentage) for categorical variables. We calculated 95% CIs surrounding the median using the bootstrap method through the boot package in R statistical software set to 10 000 iterations.

Protein and amino acid intake (milligrams per kilogram body weight per·day) were compared with Recommended Dietary Allowances from Dietary Reference Intakes.^18^ Nutrient adequacy ratios were calculated by dividing nutrient intake by its recommendation (with scores being truncated at 1) for each participant and then finding the mean of values across the entire sample for each nutrient.^19^ The mean adequacy ratio was calculated by taking the mean of all nutrient adequacy ratios.^19^ We obtained 95% CIs via bootstrapping (see previous description).

The median protein and amino acid contribution of individual food items was calculated, and the top 30 or 10 food items for protein and essential amino acids, respectively, were plotted. To further assess potential factors associated with inadequate protein intake, participants were classified as having inadequate (<0.8 g/kg) or adequate (≥0.8 g/kg) protein intake,^18^ and logistic regression models with Firth penalization^20^ were used considering protein intake status as the outcome variable. Protein supplements or texturized soy protein consumption were used as binary variables (yes or no) in the main analysis. Quartiles were calculated for continuous variables (eg, unprocessed and minimally processed food, ultraprocessed food, and unprocessed and ultraprocessed protein intake) and used as categorical variables. Models were adjusted for age, sex, body weight, and energy intake relative to body weight. The α level was set at .05 for all analyses, and all *P* values assume a 2-sided test. All data cleaning, exploration, and visualization was performed using R statistical software version 4.2.2 (R Project for Statistical Computing) and RStudio (Posit Software, PBC) with dplyr and ggplot2 packages.

## Results

Among 1014 participants who completed the survey, 43 individuals were excluded for not fully adhering to a vegan diet and 197 individuals were excluded due to insufficient report on foods and portions, resulting in 774 individuals (median [IQR] age, 29 [24-35] years; 637 female [82.3%]) who completed the survey, were confirmed as adhering to a vegan diet, and provided adequate food recalls. The median body mass index (BMI; calculated as weight in kilograms divided by height in meters squared) was 22.6 (20.3-24.8). Most participants were from the Southeast region of Brazil (491 participants [63.4%]), had a high educational level (280 participants with postgraduate education [36.2%]), were within socioeconomic class B (296 participants [38.2%]), reported no alcohol consumption (324 participants [41.9%]) or smoking habits (706 participants [91.2%]), exercised from 5 to 6 hr/wk (176 participants [22.7%]), and had adhered to a vegan diet for 5 years or longer (219 participants [28.3%]) (Table 1). Because 216 participants did not report body weight, relative protein and amino acid intakes were available for 558 individuals (complete case population; median [IQR] age, 29 [24-37] years; 459 female [82.3%]) (see eFigure 1 in Supplement 1 for study flowchart and eTable 2 in Supplement 1 for all demographic information).

**Table 1.** Study Population Characteristics

Characteristic	Participants, No. (%)		
	Overall (n = 774)	Female (n = 637)	Male (n = 137)
Age, median (IQR), y	29 (24-35)	28 (24-35)	31 (25-35)
Anthropometric measures			
No. with data^a^	558	459	99
Body weight, median (IQR), kg	60 (54-71)	59 (53-66)	74 (65-82)
Height, median (IQR), cm	165 (160-170)	163 (159-168)	175 (170-180)
BMI, median (IQR)	22.6 (20.3-24.8)	22.1 (20.1-24.3)	23.9 (22.7-25.9)
Region			
Central-West	43 (5.6)	34 (5.3)	9 (6.6)
North	4 (0.5)	4 (0.6)	0 (0.0)
Northeast	70 (9.0)	56 (8.8)	14 (10.2)
South	166 (21.4)	144 (22.6)	22 (16.1)
Southeast	491 (63.4)	399 (62.6)	92 (67.2)
Educational level			
Elementary school, incomplete	2 (0.3)	2 (0.3)	0 (0.0)
Elementary school, completed	2 (0.3)	1 (0.2)	1 (0.7)
High school, incomplete	18 (2.3)	18 (2.8)	0 (0.0)
High school, completed	55 (7.1)	50 (7.8)	5 (3.6)
Undergoing college or technician education	175 (22.6)	136 (21.4)	39 (28.5)
College education or technician, complete	242 (31.3)	190 (29.8)	52 (38.0)
Postgraduate	280 (36.2)	240 (37.7)	40 (29.2)
Income class^b^			
A	41 (5.3)	29 (4.6)	12 (8.8)
B	296 (38.2)	243 (38.1)	53 (38.7)
C	196 (25.3)	161 (25.3)	35 (25.5)
D or E	241 (31.1)	204 (32.0)	37 (27.0)
Smoking status (smoker)	68 (8.8)	53 (8.3)	15 (10.9)
Alcohol consumption			
None	324 (41.9)	267 (41.9)	57 (41.6)
1-2 Times/mo	147 (19.0)	127 (19.9)	20 (14.6)
2-3 Times/wk	72 (9.3)	60 (9.4)	12 (8.8)
2-4 Times/mo	222 (28.7)	176 (27.6)	46 (33.6)
≥4 Times/wk	9 (1.2)	7 (1.1)	2 (1.5)
Habitual physical exercise, h/wk			
Does not exercise	142 (18.3)	126 (19.8)	16 (11.7)
1-2	156 (20.2)	136 (21.4)	20 (14.6)
3-4	176 (22.7)	145 (22.8)	31 (22.6)
5-6	176 (22.7)	142 (22.3)	34 (24.8)
≥7	124 (16.0)	88 (13.8)	36 (26.3)
How long as a vegan, y			
<1	110 (14.2)	89 (14.0)	21 (15.3)
1-2	174 (22.5)	141 (22.1)	33 (24.1)
2-3	153 (19.8)	127 (19.9)	26 (19.0)
3-4	118 (15.2)	102 (16.0)	16 (11.7)
≥5	219 (28.3)	178 (27.9)	41 (29.9)
Supplement use	110 (14.2)	89 (14.0)	21 (15.3)
Nutritional intake, median (IQR)			
Total, kcal	1782 (1385-2227)	1725 (1365-2127)	2200 (1729-2914)
Protein, g	70 (48-94)	67 (48-89)	86 (60-126)
Carbohydrate, g	268 (204-346)	255 (198-325)	342 (261-439)
Fat, g	53 (37-72)	51 (36-67)	62 (44-88)
Protein relative to body weight^a^			
No. with data	558	459	99
g/kg	1.12 (0.79-1.53)	1.11 (0.79-1.48)	1.14 (0.78-1.69)
Proportion of total energy intake, %			
Carbohydrate	58.9 (52.3-64.7)	58.6 (52.0-64.5)	60.2 (55.1-65.0)
Protein	14.9 (12.3-18.8)	14.9 (12.2-18.8)	14.7 (12.2-18.8)
Fat	24.7 (18.8-30.8)	24.9 (18.7-30.9)	23.7 (19.2-29.1)
Saturated fatty acid, g	10 (7-14)	9 (6-14)	12 (8-19)
Monounsaturated fatty acid, g	19 (13-28)	18 (12-26)	23 (16-35)
Polyunsaturated fatty acid, g	16 (11-22)	15 (10-21)	20 (11-28)
Trans-fatty acid, g	0.04 (0.01-0.08)	0.04 (0.01-0.08)	0.06 (0.02-0.13)
Dietary fiber, g	44 (31-61)	41 (30-57)	58 (39-76)

Abbreviation: BMI, body mass index (calculated as weight in kilograms divided by height in meters squared).

^a^ This value is among the 558 participants who provided body weight and height data.

^b^ Income was categorized according to the Brazilian Institute of Geography and Statistics in classes A (≥US $4200), B (US $1350-$4200), C (US $550-$1350), and D or E (≤US $550).^12^

**Figure 1.**
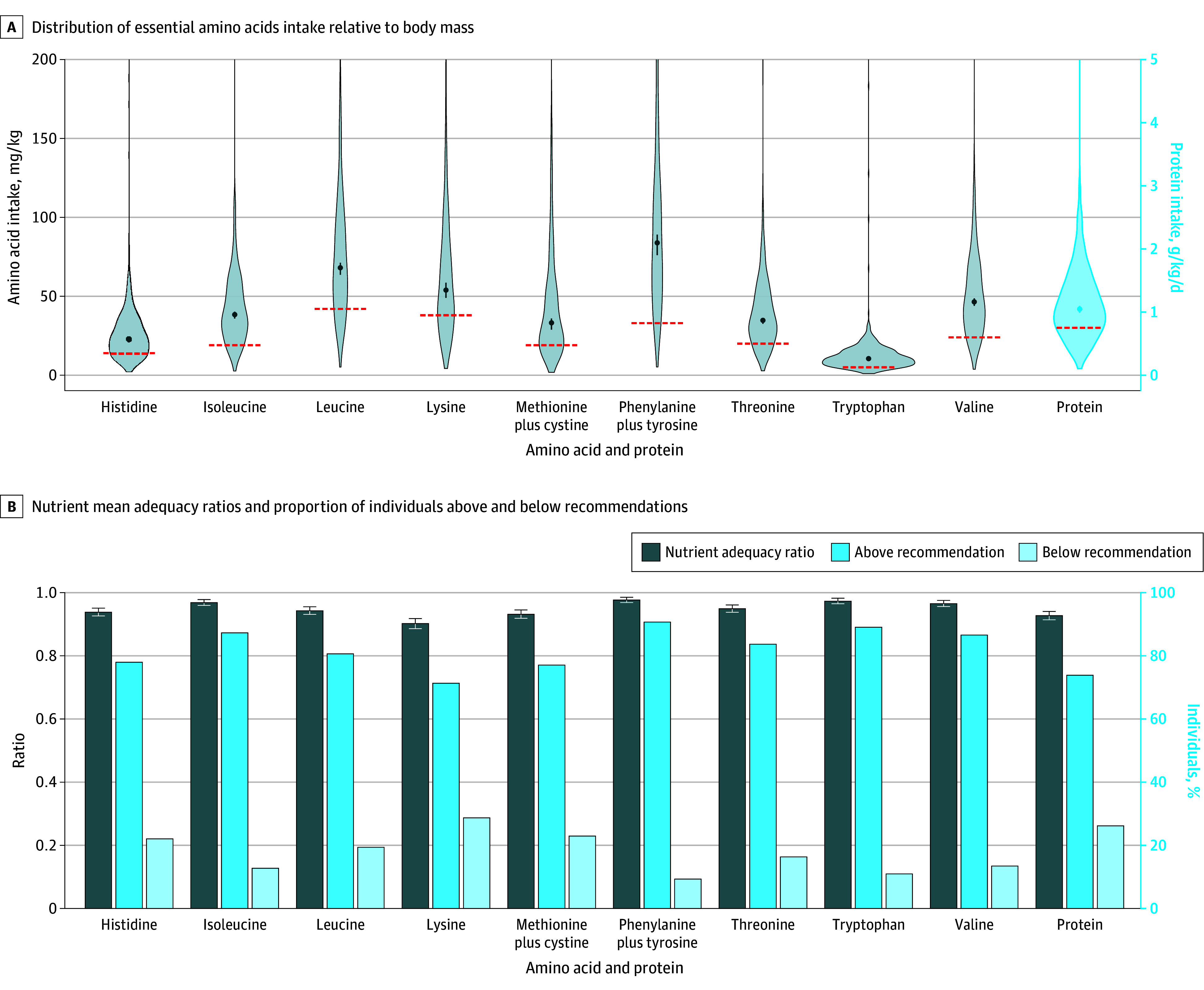
Nutrient Intake and Adequacy Ratios and Proportion Meeting Recommendations A, Violin plots show the distribution of essential amino acid and protein intake relative to body mass. Dots indicate medians; whiskers, 95% CIs; red dashed lines, respective recommendations. B, Nutrient mean adequacy ratios (presented as ratios, ranging from 0-1, left axis) and the proportion of individuals above and below recommendations for essential amino acids and protein (presented as percentages, ranging from 0%-100%, right axis) are shown.

In the 1-day food diary among all participants, the median (IQR) intake was 1782 (1.385-2.227) kilocalories, with a distribution of 58.9% (52.3%-64.7%) carbohydrates, 14.9% (12.3%-18.8%) proteins, and 24.7% (18.8%-30.8%) fat. The median protein intake among the complete case population was 1.12 g/kg/d (95% CI, 1.05-1.16 g/kg/d) (Figure 1, A), whereas the median (IQR) dietary fiber intake was 44 (31-61) g/d (Table 1). Other micronutrient intake levels can be found in eTable 3 in Supplement 1.

Among the complete case population, the nutrient adequacy ratio for protein intake was 0.93 (95% CI, 0.921-0.94). Median intake levels of all essential amino acids were greater than recommended levels (Figure 1, B; see eTable 4 in Supplement 1 for numerical values). Nutrient adequacy ratios were higher than 0.90 for all essential amino acids. Lysine presented the lowest (0.90 [95% CI, 0.89-0.92]), and phenylalanine and tyrosine the highest (0.98 [95% CI, 0.97-0.99]) adequacy ratios (Table 2; Figure 1, B). The mean adequacy ratio across protein and all essential amino acids was 0.95 (95% CI, 0.94-0.96). Median intake levels of nonessential amino acids are available in eTable 5 in Supplement 1.

**Table 2.** Caloric Contribution and Protein Intake by Nova Food Processing Category and Amino Acid Intake

Category	Outcome, median (95% CI)^a^		
**Intake by processing category**			
No. with data	774	637	137
Caloric contribution, % total energy intake			
Unprocessed and minimally processed food	66.5 (65.0-67.9)	66.4 (64.7-68.0)	67.0 (64.5-69.6)
Processed culinary ingredients	8.3 (7.6-8.8)	8.4 (7.7-9.0)	8.1 (6.6-9.4)
Processed food	6.2 (5.1-6.8)	6.6 (5.7-7.5)	3.9 (0.8-6.7)
Ultraprocessed food	13.2 (12.4-14.4)	13.2 (12.3-14.4)	13.1 (10.1-16.0)
Protein intake, % total protein intake			
Unprocessed and minimally processed food	61.8 (59.4-64.0)	61.8 (58.7-64.6)	62.2 (57.3-66.2)
Processed food	7.4 (6.1-8.7)	7.8 (6.4-9.2)	5.2 (2.5-9.3)
Ultraprocessed food	23.6 (21.0-26.3)	22.9 (20.0-26.8)	26.1 (21.7-30.6)
**Protein and essential amino acid intake by body weight**			
No. with data	558	459	99
Protein, g/kg/d	1.12 (1.05-1.16)	1.11 (1.04-1.16)	1.13 (0.86-1.30)
Essential amino acids, mg/kg			
Tryptophan	10.5 (10.1-11.1)	10.6 (10.1-11.3)	10.2 (7.5-11.3)
Threonine	34.8 (32.6-36.2)	34.8 (32.6-36.3)	34.7 (21.3-38.6)
Isoleucine	38.4 (35.9-40.2)	38.3 (36.3-40.1)	41.6 (35.3-51.0)
Leucine	68.1 (63.7-71.3)	67.6 (62.8-70.3)	69.5 (44.2-77.1)
Lysine	53.9 (49.0-58.5)	52.8 (47.7-56.8)	57.3 (41.8-69.9)
Methionine	15.5 (13.9-16.5)	15.2 (13.8-16.3)	18.7 (14.1-23.1)
Cystine	16.9 (15.3-18.8)	16.1 (14.2-17.6)	21.8 (17.1-28.7)
Methionine and cystine	33.2 (28.8-35.9)	32.4 (29.3-34.8)	41.0 (32.7-51.5)
Phenylalanine	51.5 (48.3-54.5)	50.6 (46.6-53.2)	53.6 (37.0-63.2)
Tyrosine	32.0 (29.2-34.5)	31.2 (28.6-33.6)	39.3 (33.5-49.4)
Phenylalanine and tyrosine	83.9 (75.2-89.1)	82.4 (74.1-87.6)	100.6 (83.1-131.2)
Valine	46.5 (44.0-48.9)	46.4 (44.0-48.8)	47.6 (33.4-55.7)

^a^ Results are presented as medians with 95% CIs, which show the estimated range containing the true population median for each variable, with 95% confidence.

**Figure 2.**
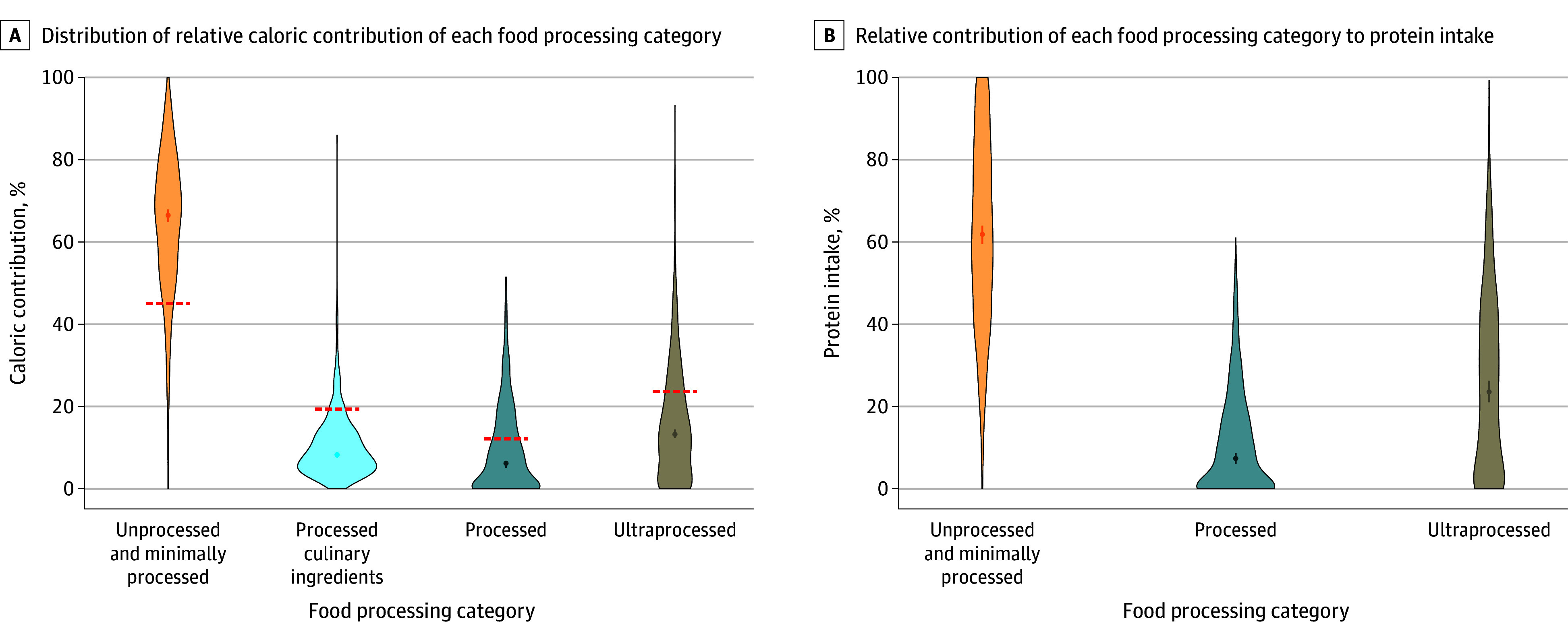
Caloric and Protein Intake by Nova Food Processing Category A, Violin plots show the distribution of relative caloric contribution of each food processing category. Dashed lines indicate reference values from the Brazilian population living in metropolitan areas. B, The relative contribution of each food processing category to protein intake is shown. Dots indicate medians; whiskers, 95% CIs.

Among the full study population, there was a high intake level of unprocessed and minimally processed food (66.5% [95% CI, 65.0%-67.9%] of total energy intake), which was higher than values reported for metropolitan areas in Brazil.^21^ They had a low intake level as a percentage of total energy intake of processed culinary ingredients (8.3% [95% CI, 7.6%-8.8%]), processed food (6.2% [95% CI, 5.1%-6.8%]), and ultraprocessed food (13.2% [95% CI, 12.4%-14.4%]), all lower than values reported in metropolitan areas in Brazil (Table 2; Figure 2).^21^ Unprocessed and minimally processed food were also the main source of protein as the percentage of total energy from proteins (61.8% [95% CI, 59.4%-64.0%]), followed by ultraprocessed (23.6% [95% CI, 21.0%-26.3%]) and processed (7.4% [95% CI, 6.1%-8.7%]) foods. In a sensitivity analysis considering textured soy protein as unprocessed and minimally processed rather than ultraprocessed food, there was a decrease in the contribution of ultraprocessed food to total calories from 13.2% to 10.7% (95% CI, 9.4%-11.7%) and energy intake from proteins from 23.6% to 14.3% (95% CI, 12.2%-16.0%) (eTable 6 and eFigure 2 in Supplement 1). Individual food analyses for the main sources of protein and essential amino acids are available in eFigure 3 in Supplement 1.

**Figure 3.**
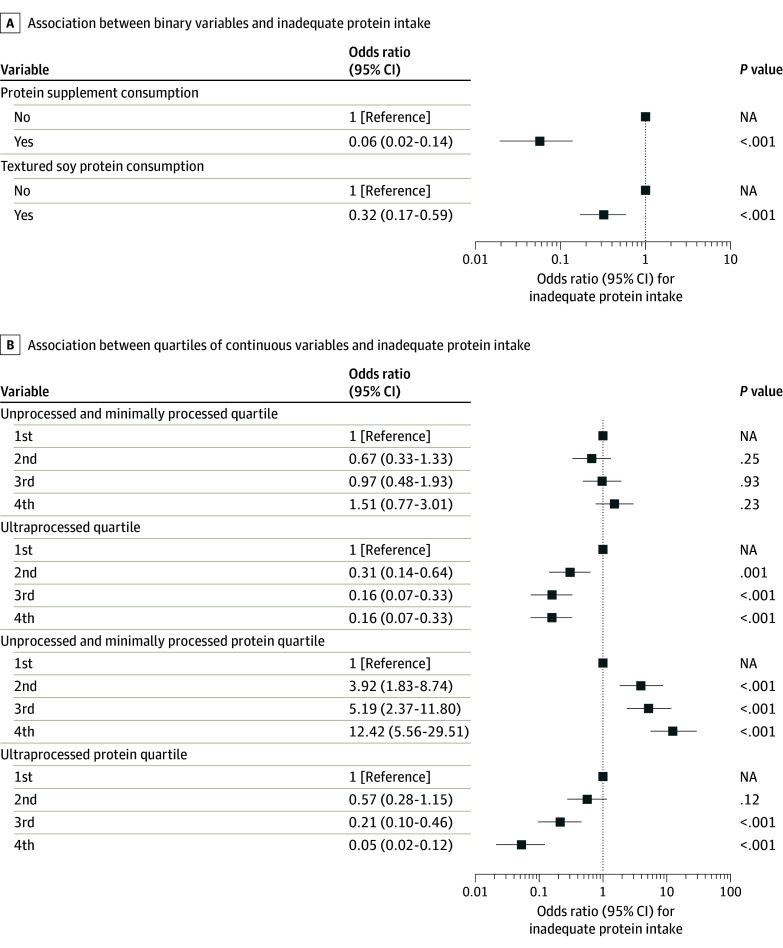
Logistic Regression Models for Association of Variables With Protein Inadequacy A, The association between binary variables (consuming protein supplements and consuming textured soy protein) with inadequate protein intake is shown. B, The association between quartiles of continuous variables and inadequate protein intake is shown. Results are presented as odds ratio coefficients and 95% CIs for having inadequate protein intake given the variable in comparison with the reference level. NA indicates not applicable.

Adjusted logistic regression models among the complete case population showed that consuming protein supplements (odds ratio [OR], 0.06 [95% CI, 0.02-0.14]; *P* < .001) and textured soy protein (OR, 0.32 [95% CI, 0.17-0.59]; *P* < .001) compared with not consuming those products was associated with reduced odds of displaying inadequate protein intake (Figure 3A). The second, third, and fourth quartiles of ultraprocessed food intake (eg, fourth vs first quartile of intake: OR, 0.16 [95% CI, 0.07-0.33]; *P* < .001) and the third and fourth quartiles of ultraprocessed protein intake were associated with reduced odds of displaying inadequate protein intake, while the second, third, and fourth quartiles of unprocessed protein intake (eg, fourth vs first quartile of intake: OR, 12.42 [95% CI, 5.56-29.51]; *P* < .001) was associated with increased odds of displaying inadequate protein intake compared with the respective first quartile (see Figure 3B for model coefficients and eTable 7 in Supplement 1 for quartiles). Results remained virtually unchanged in analyses performed with the imputed dataset (774 participants), not altering the original interpretation of our results (eTable 8 and eFigure 4 in Supplement 1). An exploratory analysis considering protein supplement and texturized soy protein intake as continuous rather than binary variables showed a negative association of protein supplement and texturized soy protein intake with the probability of displaying protein inadequacy (eFigure 5 in Supplement 1), which is in agreement with our main analysis.

## Discussion

This cross-sectional survey study analyzed self-reported data from 1-day food diaries on protein and amino acid intake and food processing level among individuals who adhered to a vegan diet. The main findings were that the mean adequacy ratio for protein and essential amino acids was high, participants had a high level of consumption of unprocessed and minimally processed food and a low level of consumption of processed and ultraprocessed food, and consumption of ultraprocessed protein sources was associated with decreased odds of showing inadequate protein intake, while the opposite was true for unprocessed and minimally processed protein sources.

Although previous evidence from smaller samples indicated that vegan diets may provide adequate protein, albeit lower than that of omnivorous diets,^4^ it was still unclear whether they delivered enough essential amino acids. In our sample, a high mean adequacy for protein and essential amino acids was observed. To the best of our knowledge, the largest previous study to address amino acid intake in vegans was a cross-sectional analysis of the European Prospective Investigation into Cancer and Nutrition (EPIC)–Oxford cohort (98 individuals).^22^ The authors found lower amino acid intake among individuals who adhered to a vegan diet compared with meat-eaters; however, whether this difference was associated with relevant deficiencies was unknown.^22^ Based on a much larger sample and using a higher-resolution instrument, our study offers novel evidence that the most vegans can meet essential amino acid recommendations.

However, a considerable segment of participants did not meet recommendations for particular essential amino acids. For instance, lysine and the combination of methionine and cystine showed the lowest nutrient adequacy in our sample. Indeed, these amino acids are generally lower in plant- vs animal-based proteins.^23,24^ Importantly, all amino acids are needed in adequate amounts to support de novo tissue protein synthesis,^25^ reinforcing the need for properly planned, well-balanced combinations of different plant-based protein sources to attain an optimal essential amino acid profile.

Mounting evidence shows that increased ultraprocessed food consumption favors several negative health-related outcomes, such as higher energy intake and weight gain, higher prevalence of nontransmissible chronic diseases, and an overall increased mortality rate.^26,27,28,29,30,31^ Although vegan diets are traditionally based on fresh and in natura foods, there has been a tendency of increased ultraprocessed food consumption in vegans due to the proliferation of plant-based meat and dairy substitutes, possibly compromising the quality of vegan diets.^5^ A previous study in a French cohort^6^ found ultraprocessed food consumption to be 39.5% of total energy intake among individuals who adhered to a vegan diet vs 33% in meat-eaters. In our study, unprocessed and minimally processed foods represented the highest caloric contribution in vegan diets, with a smaller presence of ultraprocessed foods. One may argue that compared with other countries, the plant-based meat and dairy substitute industry in Brazil may be less active, providing fewer or less attractive options. Considering the global trend in the protein market toward an increasing popularity of plant-based protein and the growing demand for high-quality, natural, and sustainable protein sources,^32^ an increase in consumption of ultraprocessed foods among Brazilian individuals who adhere to a vegan diet may be expected in the future, warranting follow-up surveys to monitor possible health outcomes.

Recently, the discussion on whether all subgroups of ultraprocessed foods are equally harmful compared with one another has gained traction. Indeed, a 2023 study^33^ found that distinct subgroups of ultraprocessed foods were differently associated with type 2 diabetes risk; subgroups, such as fruit- and dairy-based ultraprocessed desserts, had a risk reduction. Another study^34^ found that although ultraprocessed food consumption was associated with multimorbidity related to cancer and cardiometabolic diseases, this association was not seen in the subgroup of plant-based ultraprocessed foods, suggesting that Nova may not capture possible nuance in quality among ultraprocessed items. In our study, textured soy protein was an important contributor to ultraprocessed food consumption. This is a challenging food item to classify given that there is significant variability in formulations considering the presence of food additives and cosmetics. This prompted us to use a more conservative approach of classifying this food as ultraprocessed, which is in accordance with previous research.^6^ In a sensitivity analysis considering textured soy protein as unprocessed and minimally processed, the energy contribution from ultraprocessed food decreased greatly. The reduction in relative contribution of ultraprocessed food to protein intake was even more pronounced, clearly suggesting that reclassification of individual foods may be associated with impactful changes in the contribution of ultraprocessed foods to vegan diets.

We found that protein supplement, textured soy protein, ultraprocessed food, and ultraprocessed protein intake were associated with reduced probabilities of protein inadequacy. Conversely, unprocessed and minimally processed protein intake was associated with an increased probability of protein inadequacy. This may be partially explained by the lower energy and protein density in plant- vs animal-derived foods,^35^ suggesting that it may be challenging for vegans fully avoiding ultraprocessed foods to reach higher levels of protein intake without substantially increasing food (and perhaps calorie) intake. While these findings do not imply that ultraprocessed foods are essential for individuals who adhere to a vegan diet to meet protein recommendations, it reveals a significant reliance on these foods to attend protein requirements. One may suggest that certain ultraprocessed foods, such as textured soy protein, may be recommended for this population. Despite convincing data associating the broad category of ultraprocessed with poor outcomes, it is hard to reconcile textured soy protein as having detrimental health outcomes, with ample evidence suggesting otherwise.^36,37,38^ This holds true for protein supplements, an evidence-based strategy to support muscle health^39^ also associated with protein adequacy in this study. At least regarding individuals who adhere to a vegan diet, unrestricted advice to avoid ultraprocessed foods may have unintended consequences, such as protein intake inadequacies, that warrant further investigation. This also suggests that vegans may benefit from public policies aimed to facilitate access to more natural and healthy foods and amplify nutritional support and education for adequacy of overall food intake. Simultaneously, our data reinforce the urgent need for the development of affordable, healthier, better-quality, cleaner-label, and protein-rich plant-based food options by the industry.

### Strengths and Limitations

Strengths of this study include the large sample size and use of food diaries to quantify food intake.^40^ The study also has several limitations, including the cross-sectional design, self-reporting of information, and use of a 1-day food diary instead of repeated measurements of food intake. Our study features a convenience sample, predominantly composed of females, with eutrophic BMI and a high educational level. While this may limit the generalizability of our findings, epidemiological studies confirm that this as the typical sociodemographic profile of vegans.^41,42,43^ Nonetheless, our conclusions cannot be extrapolated to more population samples with a lower socioeconomical status or with different dietary patterns. Further studies are warranted to answer such questions.

## Conclusions

In this cross-sectional survey study including a 1-day dietary assessment, individuals who adhered to a vegan diet mostly attained protein and essential amino acid intake recommendations and had a lower intake of ultraprocessed food compared with previous reports on vegans and the overall Brazilian population. Importantly, ultraprocessed protein sources were associated with a decreased likelihood of inadequate protein intake, while the opposite was true for unprocessed protein sources. The role of ultraprocessed foods in vegan diets needs to be further investigated given that common protein sources in the vegan diet may not be associated with the same detrimental health outcomes as other ultraprocessed foods, while contributing to protein requirements in this population.

## Notes

Supplement 1. **eTable 1.** Examples of common vegan foods and their classification according to Nova system **eTable 2.** Comparison of subsets with available and missing data across variables **eTable 3.** Micronutrients intake **eTable 4.** Nutrient adequacy ratios of protein and essential amino acids **eTable 5.** Intake of nonessential amino acids **eTable 6.** Caloric contribution and protein intake according to Nova food processing category and amino acid intake considering textured soy protein as unprocessed and minimally processed food **eTable 7.** Coefficients from adjusted logistic regression models estimating protein inadequacy (complete case analysis, n=558) **eTable 8.** Coefficients from adjusted logistic regression models estimating protein inadequacy (imputed dataset, n=774) **eFigure 1.** Study flowchart **eFigure 2.** Caloric and protein intake according to Nova food processing categories considering textured soy protein as unprocessed and minimally processed **eFigure 3.** Main food sources of protein and essential amino acids **eFigure 4.** Protein and essential amino acids intake, nutrient adequacy ratios and proportion of individuals meeting recommended intakes in the imputed (n=774) dataset **eFigure 5.** Exploratory logistic regression models estimating the probability of protein inadequacy according to protein supplement intake or texturized soy protein intake as continuous variables

Supplement 2.
Data Sharing Statement
