# Supplementary material for: Protein and Amino Acid Adequacy and Food Consumption by Processing Level in Vegans in Brazil
Source: JAMA Netw Open. 2024 Jun 24;7(6):e2418226. doi: 10.1001/jamanetworkopen.2024.18226 (PMC11197455; doi:10.1001/jamanetworkopen.2024.18226)
Supplement: Supplement 1. — eTable 1. Examples of common vegan foods and their classification according to Nova system eTable 2. Comparison of subsets with available and missing data across variables eTable 3. Micronutrients intake eTable 4. Nutrient adequacy ratios of protein and essential amino acids eTable 5. Intake of nonessential amino acids eTable 6. Caloric contribution and protein intake according to Nova food processing category and amino acid intake considering textured soy protein as unprocessed and minimally processed food eTable 7. Coefficients from adjusted logistic regression models estimating protein inadequacy (complete case analysis, n=558) eTable 8. Coefficients from adjusted logistic regression models estimating protein inadequacy (imputed dataset, n=774) eFigure 1. Study flowchart eFigure 2. Caloric and protein intake according to Nova food processing categories considering textured soy protein as unprocessed and minimally processed eFigure 3. Main food sources of protein and essential amino acids eFigure 4. Protein and essential amino acids intake, nutrient adequacy ratios and proportion of individuals meeting recommended intakes in the imputed (n=774) dataset eFigure 5. Exploratory logistic regression models estimating the probability of protein inadequacy according to protein supplement intake or texturized soy protein intake as continuous variables [file jamanetwopen-e2418226-s001.pdf]

Supplemental Online Content

Leitão AE, Esteves GP, Mazzolani BC, et al. Protein and amino acid adequacy and food processing in vegan diets in Brazil. *JAMA Netw Open*. 2024;7(6):e2418226. doi:10.1001/jamanetworkopen.2024.18226

eTable 1. Examples of common vegan foods and their classification according to Nova system

eTable 2. Comparison of subsets with available and missing data across variables

eTable 3. Micronutrients intake

eTable 4. Nutrient adequacy ratios of protein and essential amino acids

eTable 5. Intake of nonessential amino acids

eTable 6. Caloric contribution and protein intake according to Nova food processing category and amino acid intake considering textured soy protein as unprocessed and minimally processed food

eTable 7. Coefficients from adjusted logistic regression models estimating protein inadequacy (complete case analysis, n=558)

eTable 8. Coefficients from adjusted logistic regression models estimating protein inadequacy (imputed dataset, n=774)

eFigure 1. Study flowchart

eFigure 2. Caloric and protein intake according to Nova food processing categories considering textured soy protein as unprocessed and minimally processed

eFigure 3. Main food sources of protein and essential amino acids

eFigure 4. Protein and essential amino acids intake, nutrient adequacy ratios and proportion of individuals meeting recommended intakes in the imputed (n=774) dataset

eFigure 5. Exploratory logistic regression models estimating the probability of protein inadequacy according to protein supplement intake or texturized soy protein intake as continuous variables

This supplemental material has been provided by the authors to give readers additional information about their work.

© 2024 Leitão AE et al. *JAMA Network Open.*

eTable 1. Examples of common vegan foods and their classification according to Nova system.

**Nova food classification**	**Commonly reported foods**
Unprocessed and minimally processed	Avocado<br>Chayote, Boiled with Salt, Drained<br>Chestnut, Dried<br>Chickpeas, Beans and Peas<br>Cocoa powder, unsweetened<br>Corn, Dry<br>Cornstarch<br>Cranberry, low bush or lingenberry, fresh<br>Dates, Dry<br>Eggplant<br>Flour, All Purpose Wheat, Self-Rising, Enriched<br>Flour, chickpea or besan<br>Flour, corn, white, whole grain<br>Mushrooms, boiled, drained<br>Oat Bran, Dry<br>Onions, Chopped<br>Papaya<br>Parsley, Chopped<br>Peanut Butter, Smooth<br>Pearl tapioca, dry<br>Pepper, Black, Ground<br>Popcorn, popped in oil<br>Potatoes baked<br>Quinoa<br>Rice, white, long grain, boiled<br>Seeds of sunflower, linseed, and pumpkin, whole<br>Seeds, chia, dried<br>Sesame Butter or tahini<br>Swiss Chard<br>Tea, Herbal, Prepared<br>Tempeh or Tempe<br>Tomatoes, Red
	Oil, olive

© 2024 Leitão AE et al. *JAMA Network Open.*

Nova food classification	Commonly reported foods
Processed culinary ingredients	Oil, Soybean<br>Salt, table<br>Sugar, white granulated<br>Syrup, corn or sugar<br>Vegan Butter
Processed foods	Bread, French<br>Bread, wheat<br>Candy Bar, Peanut<br>Cereal, Granola, Prepared<br>Crackers, Rice<br>Crackers, water biscuits<br>Jelly<br>Pasta, made without egg, cooked<br>Tofu, firm, with calcium and magnesium chloride
Ultra-processed foods	Acai berry drink<br>Almond milk<br>Bread, Mixed Grain<br>Cereal Bar, Rice and Wheat<br>Cheese, imitation<br>Cookie<br>Crackers, whole wheat<br>Pea protein supplements<br>Pizza<br>Potatoes or French Fries<br>Sauce, Soy (Shoyu)<br>Soda, cola<br>Soy protein - Supplements<br>Soymilk, Calcium Fortified<br>Textured Soy Protein<br>Vegan Nuggets, Baked<br>Vegan Protein Sausages<br>Veggie Burger, Unprepared<br>Yogurt, Tofu<br>Vegan Mayo

© 2024 Leitão AE et al. *JAMA Network Open.*

eTable 2. Comparison of subsets with available and missing data across variables.

	**Participants, median (interquartile range)**			
**Characteristic**	**Sample with adequate dietary reports,** N = 774	**Subset with available body weight,** N = 558	**Subset with missing body weight,** N = 216	**Subset excluded due to inadequate dietary reports,** N=197
Age	29 (24-35)	29 (24-37)	28 (23-33)	32 (25-39)
Sex				
Female, No. (%)	637 (82.3%)	459 (82.3%)	178 (82.4%)	163 (82.7%)
Male, No. (%)	137 (17.7%)	99 (17.7%)	38 (17.6%)	34 (17.3%)
Body weight (kg)	60 (54-71)	60 (54-71)	-	64 (56-70)
Height (cm)	165 (160-170)	165 (160-170)	-	166 (160-172)
BMI [a] (g/kg²)	23 (20-25)	23 (20-25)	-	23 (20-25)
**Region**				
Central-West	43 (5.6%)	29 (5.2%)	14 (6.5%)	6 (3.1%)
North	4 (0.5%)	2 (0.4%)	2 (0.9%)	2 (1.0%)
Northeast	70 (9.0%)	43 (7.7%)	27 (12.5%)	18 (9.2%)
South	166 (21.4%)	122 (21.9%)	44 (20.4%)	50 (25.5%)
Southeast	491 (63.4%)	362 (64.9%)	129 (59.7%)	121 (61.7%)
**Educational level, No. (%)**				
Elementary school, incomplete	2 (0.3%)	1 (0.2%)	1 (0.5%)	4 (2.0%)
Elementary school, completed	2 (0.3%)	1 (0.2%)	1 (0.5%)	1 (0.5%)
High school, incomplete	18 (2.3%)	9 (1.6%)	9 (4.2%)	4 (2.0%)
High school, completed	55 (7.1%)	32 (5.7%)	23 (10.6%)	20 (10.2%)

© 2024 Leitão AE et al. *JAMA Network Open.*

Characteristic	Participants, median (interquartile range) Sample with adequate dietary reports, N = 774	Subset with available body weight, N = 558	Subset with missing body weight, N = 216	Subset excluded due to inadequate dietary reports, N=197
Undergoing college or technician education	175 (22.6%)	120 (21.5%)	55 (25.5%)	47 (23.9%)
College education or technician, complete	242 (31.3%)	176 (31.5%)	66 (30.6%)	47 (23.9%)
Postgraduate	280 (36.2%)	219 (39.2%)	61 (28.2%)	74 (37.6%)
**Income, No. (%)**				
A class	41 (5.3%)	23 (4.1%)	18 (8.3%)	9 (4.6%)
B class	296 (38.2%)	222 (39.8%)	74 (34.3%)	63 (32.0%)
C class	196 (25.3%)	147 (26.3%)	49 (22.7%)	56 (28.4%)
D/E class	241 (31.1%)	166 (29.7%)	75 (34.7%)	69 (35.0%)
**Smoking status, No. (%)**	68 (8.8%)	45 (8.1%)	23 (10.6%)	26 (13.2%)
**Alcohol consumption, No. (%)**				
No alcohol consumption	324 (41.9%)	236 (42.3%)	88 (40.7%)	81 (41.1%)
Once to twice a month	147 (19.0%)	108 (19.4%)	39 (18.1%)	33 (16.8%)
Twice to three times per week	72 (9.3%)	46 (8.2%)	26 (12.0%)	28 (14.2%)
Twice to four times a month	222 (28.7%)	162 (29.0%)	60 (27.8%)	51 (25.9%)
Four or move times per week	9 (1.2%)	6 (1.1%)	3 (1.4%)	4 (2.0%)

© 2024 Leitão AE et al. *JAMA Network Open.*

Characteristic	Participants, median (interquartile range) Sample with adequate dietary reports, N = 774	Subset with available body weight, N = 558	Subset with missing body weight, N = 216	Subset excluded due to inadequate dietary reports, N=197
**Habitual physical exercise, No. (%)**				
Does not exercise	142 (18.3%)	108 (19.4%)	34 (15.7%)	52 (26.4%)
1-2 hour/week	156 (20.2%)	111 (19.9%)	45 (20.8%)	55 (27.9%)
3-4 hour/week	176 (22.7%)	126 (22.6%)	50 (23.1%)	43 (21.8%)
5-6 hour/week	176 (22.7%)	122 (21.9%)	54 (25.0%)	28 (14.2%)
7 hour/week or more	124 (16.0%)	91 (16.3%)	33 (15.3%)	19 (9.6%)
**How long as a vegan, No. (%)**				
Less than one year	110 (14.2%)	72 (12.9%)	38 (17.6%)	25 (12.7%)
1 to 2 years	174 (22.5%)	123 (22.0%)	51 (23.6%)	50 (25.4%)
2 to 3 years	153 (19.8%)	111 (19.9%)	42 (19.4%)	30 (15.2%)
3 to 4 years	118 (15.2%)	94 (16.8%)	24 (11.1%)	31 (15.7%)
5 or more years	219 (28.3%)	158 (28.3%)	61 (28.2%)	61 (31.0%)
Supplement use	592 (76.5%)	430 (77.1%)	162 (75.0%)	113 (57.4%)
Kilocalories (kcal)	1,782 (1,385-2,227)	1,777 (1,392-2,257)	1,805 (1,356-2,225)	-
Protein (g)	70 (48-94)	69 (48-93)	72 (50-98)	-
Carbohydrate (g)	268 (204-346)	266 (204-345)	270 (205-348)	-
Fat (g)	53 (37-72)	53 (37-73)	51 (36-68)	-
Protein (g/kg)	1.12 (0.79-1.53)	1.00 (0.80-2.00)	-	-
Protein (% Total energy intake)	15 (12-19)	15 (12-18)	15 (13-20)	-
Carbohydrate (% TEI)	59 (52-65)	58 (52-65)	60 (53-65)	-

© 2024 Leitão AE et al. *JAMA Network Open.*

Characteristic	Participants, median (interquartile range)			
	**Sample with adequate dietary reports,** N = 774	**Subset with available body weight,** N = 558	**Subset with missing body weight,** N = 216	**Subset excluded due to inadequate dietary reports,** N=197
Fat (% TEI)	25 (19-31)	25 (19-31)	24 (18-29)	-
Saturated fatty acid (g)	10 (7-14)	10 (7-15)	9 (6-14)	-
Monounsatured fatty acid (g)	19 (13-28)	19 (13-28)	17 (13-26)	-
Polyunsaturated fatty acid (g)	16 (11-22)	16 (11-22)	16 (10-22)	-
Trans-fatty acid (g)	0.04 (0.01-0.08)	0 (0-0)	0 (0-0)	-
Sodium (mg)	2,376 (1,536-3,233)	2,392 (1,589-3,234)	2,281 (1,407-3,218)	-
Potassium (mg)	3,626 (2,716-4,638)	3,614 (2,692-4,629)	3,762 (2,847-4,663)	-
Vitamin A (retinol) (mcg)	869 (403-1,642)	870 (403-1,685)	867 (402-1,511)	-
Vitamin A (IU)	8,419 (3,922-16,119)	8,419 (3,987-16,487)	8,475 (3,827-14,767)	-
Beta carotene (µg)	4,317 (1,801-8,049)	4,412 (1,804.8-8,399)	4,049 (1,786-7,286)	-
Alpha carotene (µg)	710 (96-2,102)	712 (95-2,139)	700 (98-2,073)	-
Lutein (µg)	1,979 (942-4,227)	1,998 (959-4,340)	1,967 (933-3,900)	-
Cryptoxanthin beta (µg)	129 (7-729)	151 (7-709)	79 (8-738)	-
Lycopene (µg)	1,831 (8-4,566)	1,894 (12-4,570)	1,686 (1.8-4,201)	-
Vitamin C (mg)	157 (81-248)	155 (81-248)	161 (79-247)	-
Calcium (mg)	571 (371-841)	582 (370-850)	544 (375-785)	-
Iron (mg)	17 (13-23)	17 (13-23)	17 (13-23)	-
Alpha-tocopherol (vitamin E) (mg)	9 (6-14)	9 (7-14)	9 (6-14)	-
Thiamin (mg)	1.69 (1.25-2.32)	2 (1.30-2.00)	2 (1.20-2.00)	-

© 2024 Leitão AE et al. *JAMA Network Open.*

	**Participants, median (interquartile range)**			
**Characteristic**	**Sample with adequate dietary reports,** N = 774	**Subset with available body weight,** N = 558	**Subset with missing body weight,** N = 216	**Subset excluded due to inadequate dietary reports,** N=197
Riboflavin (mg)	1.09 (0.82-1.47)	1 (0.80-1.00)	1 (0.80-1.00)	-
Niacin (mg)	14 (10-19)	14 (10-19)	15 (11-19)	-
Pyridoxine (mg)	1.82 (1.35-2.51)	2 (1.40-2.00)	2.00 (1.30-3.00)	-
Folate (mg)	703 (506-941)	701 (515-941)	704 (494-941)	-
Cobalamin (mg)	0.05 (0.00-1.78)	0.00 (0.00-2.00)	0.00 (0.0-2.00)	-
Biotin (mg)	14 (8-24)	14 (8-24)	13 (8-24)	-
Pantothenic (mg)	4.12 (3.20-5.73)	4.00 (3.2-6.00)	4 (3.30-6.00)	-
Vitamin K (µg)	122 (72-215)	121 (72-212)	131 (68-216)	-
Phosphorus (mg)	1,130 (803-1,509)	1,123 (808-1,503)	1,142 (797-1,542)	-
Magnesium (mg)	442 (329-594)	440 (332-602)	444 (321-587)	-
Zinc (mg)	8.2 (5.9-10.8)	8.0 (5.9-11.0)	8 (5.8-11.0)	-
Copper (mg)	1.95 (1.42-2.65)	2.00 (1.50-3.00)	2.00 (1.30-3.00)	-
Manganese (mg)	6.0 (4.2-8.5)	6.0 (4.2-9.0)	6.0 (4.3-8.0)	-
Selenium (µg)	61 (44-84)	60 (43-83)	62 (46-84)	-
Fluoride (µg)	198 (74-377)	197 (71-383)	200 (94-342)	-
Chromium (mg)	0.05 (0.03-0.09)	0.00 (0.00-0.00)	0.00 (0.00-0.00)	-
Molybdenum (mg)	5 (1-17)	5 (1-17)	5 (1-15)	-
Choline (mg)	199 (145-263)	199 (143-262)	199 (147-264)	-
Dietary fiber (g)	44 (31-61)	44 (31-60)	43 (30-63)	-

[a] body mass index.

© 2024 Leitão AE et al. *JAMA Network Open.*

eTable 3. Micronutrients intake.

	Nutrients, median (interquartile range)		
**Characteristic**	**Overall**, N = 774	**Female**, N = 637	**Male**, N = 137
Sodium (mg)	2,376 (1,536-3,233)	2,282 (1,486-3,106)	2,871 (1,833-4,091)
Potassium (mg)	3,626 (2,716-4,638)	3,452 (2,628-4,418)	4,389 (3,308-6,055)
Vitamin A (retinol) (mcg)	869 (403-1,642)	842 (412-1,602)	1,040 (341-1,880)
Vitamin C (mg)	157 (81-248)	154 (79-245)	172 (87-270)
Calcium (mg)	571 (371-841)	565 (357-827)	598 (407-881)
Iron (mg)	17 (13-23)	17 (12-22)	21 (15-28)
Alpha-tocopherol (vitamin E) (mg)	9 (6-14)	9 (6-13)	12 (8-17)
Thiamin (mg)	1.69 (1.25-2.32)	1.62 (1.21-2.15)	2.24 (1.61-2.89)
Riboflavin (mg)	1.09 (0.82-1.47)	1.06 (0.77-1.43)	1.28 (1.01-1.71)
Niacin (mg)	14 (10-19)	14 (10-18)	18 (14-23)
Pyridoxine (mg)	1.82 (1.35-2.51)	1.73 (1.29-2.35)	2.38 (1.66-3.22)
Folate (mg)	703 (506-941)	670 (487-887)	875 (624-1,133)
Cobalamin (mg)	0.05 (0.00-1.78)	0.06 (0.00-1.76)	0.05 (0.00-2.04)
Biotin (mg)	14 (8-24)	13 (7-23)	18 (9-33)
Pantothenic acid (mg)	4.12 (3.20-5.73)	3.95 (3.07-5.40)	5.39 (3.64-6.65)
Vitamin K (µg)	122 (72-215)	118 (70-214)	139 (82-216)
Phosphorus (mg)	1,130 (803-1,509)	1,092 (791-1,440)	1,367 (933-1,869)
Magnesium (mg)	442 (329-594)	430 (315-567)	529 (381-763)
Zinc (mg)	8.2 (5.9-10.8)	7.9 (5.7-10.2)	10.4 (6.9-13.7)
Copper (mcg)	1950 (1420-2650)	1.870 (1380-2540)	2380 (1660-3330)
Manganese (mg)	6.0 (4.2-8.5)	5.7 (4.0-7.9)	8.0 (5.0-10.8)
Selenium (µg)	61 (44-84)	59 (43-78)	79 (50-116)

Micronutrient intake reported herein does not consider the contribution from isolated micronutrient supplementation.

© 2024 Leitão AE et al. *JAMA Network Open.*

eTable 4. Nutrient adequacy ratios of protein and essential amino acids

Protein or amino acid	Nutrient adequacy ratio (95% CI)
Protein	0.93 (0.91-0.94)
Tryptophan	0.97 (0.96-0.98)
Threonine	0.95 (0.94-0.96)
Isoleucine	0.97 (0.96-0.98)
Leucine	0.94 (0.93-0.96)
Lysine	0.90 (0.89-0.92)
Methionine	-
Cystine	-
Methionine and Cystine	0.93 (0.92-0.94)
Phenylalanine	-
Tyrosine	-
Phenylalanine and Tyrosine	0.98 (0.97-0.99)
Valine	0.97 (0.96-0.98)

Nutrient adequacy ratios show the mean value across the entire sample of 558 individuals. The mean adequacy ratio across protein and all essential amino acids was 0.95 (95% CI, 0.94-0.96).

© 2024 Leitão AE et al. *JAMA Network Open.*

eTable 5. Intake of nonessential amino acids

**Nonessential amino acid intake, mg/kg**	**Overall,** N = 558	**Female,** N = 459	**Male,** N = 99
Arginine	62.5 (58.9-66.4)	61.8 (57.4-65.7)	65.3 (50.1-80.4)
Histidine	23.1 (21.3-24.4)	23.1 (21.3-24.5)	23.0 (16.3-26.6)
Alanine	40.2 (38.3-42.9)	40.7 (39.1-43.9)	38.7 (30.0-42.9)
Aspartic acid	97.0 (92.2-102.3)	96.7 (91.8-102.1)	99.4 (71.1-116.1)
Glutamic acid	163.3 (152.2-172.3)	163.1 (151.0-173.3)	164.4 (132.0-184.6)
Glycine	38.5 (36.0-40.6)	38.5 (35.7-40.7)	38.5 (28.8-45.0)
Proline	49.5 (46.1-51.9)	49.4 (46.0-51.8)	50.6 (38.5-58.7)
Serine	46.5 (44.0-49.6)	46.5 (44.0-49.3)	45.8 (35.7-53.2)

Results are presented as medians (with 95% CIs) which show the estimated range containing the true population median for each variable, with 95% confidence.

© 2024 Leitão AE et al. *JAMA Network Open.*

eTable 6. Caloric contribution and protein intake according to Nova food processing category and amino acid intake (considering textured soy protein as unprocessed and minimally processed food).

	**Nutrient intake, median (95% CI)**		
**Food processing category**	**Overall**, N = 774	**Female**, N = 637	**Male**, N = 137
Caloric contribution			
Unprocessed and minimally processed food (% total energy intake)	68.6 (67.6-70)	68.7 (67.6-70.3)	68.2 (63.5-70.8)
Processed culinary ingredients (% total energy intake)	8.3 (7.6-8.8)	8.4 (7.8-9)	8.1 (6.6-9.4)
Processed food (% total energy intake)	6.2 (5.1-6.8)	6.6 (5.7-7.5)	3.9 (0.6-6.7)
Ultra-processed food (% total energy intake)	10.7 (9.4-11.7)	10.5 (9-11.5)	11 (8.5-13.6)
Protein intake according to processing			
Unprocessed and minimally processed food (% total protein intake)	71.0 (69.2-72.6)	71.2 (69.3-73.6)	70.7 (66.3-74)
Processed food (% total protein intake)	7.4 (6.1-8.7)	7.8 (6.4-9.2)	5.2 (2.5-9.3)
Ultra-processed food (% total protein intake)	14.3 (12.2-16.0)	13.9 (11.9-16.1)	17.7 (13.3-22)

Results are presented as medians (with 95% CIs) which show the estimated range containing the true population median for each variable, with 95% confidence.

© 2024 Leitão AE et al. *JAMA Network Open.*

eTable 7. Coefficients from adjusted logistic regression models predicting protein inadequacy (complete case analysis, n=558).

Independent variable	Response	Odds ratio (95% CI)	P-value
Isolated protein supplement consumer	No	1.00 (reference)	-
	Yes	0.057 (0.02, 0.14)	<.001
Textured soy protein consumer	No	1.00 (reference)	-
	Yes	0.322 (0.17, 0.59)	<.001

Independent variable	Quartile	Value	Odds ratio (95% CI)	P-value
Unprocessed and minimally processed food	1st quartile	< 54.6 % TEI [a]	1.00 (reference)	-
	2nd quartile	67.2 % TEI	0.667 (0.33, 1.33)	.25
	3rd quartile	76.8 % TEI	0.968 (0.48, 1.93)	.93
	4th quartile	> 76.8 % TEI	1.514 (0.77, 3.01)	.23
Ultra-processed food	1st quartile	< 3.7 % TEI	1.00 (reference)	-
	2nd quartile	12.4 % TEI	0.305 (0.14, 0.64)	.001
	3rd quartile	23.0 % TEI	0.159 (0.07, 0.33)	<.001
	4th quartile	> 23.0 % TEI	0.159 (0.07, 0.33)	<.001
Unprocessed and minimally processed protein	1st quartile	< 46.0 % TEI	1.00 (reference)	-
	2nd quartile	62.7 % TEI	3.923 (1.82, 8.74)	<.001
	3rd quartile	81.2 % TEI	5.186 (2.37, 11.80)	<.001
	4th quartile	> 81.2 % TEI	12.415 (5.55, 29.51)	<.001
Ultra-processed protein	1st quartile	< 5.1 % TEI	1.00 (reference)	-
	2nd quartile	22.0% TEI	0.566 (0.27, 1.15)	.12
	3rd quartile	41.1 % TEI	0.214 (0.10, 0.46)	<.001
	4th quartile	> 41.1 % TEI	0.053 (0.02, 0.12)	<.001

All models were adjusted for body weight, sex, age and energy intake relative to body weight. [a] TEI = total energy intake.

© 2024 Leitão AE et al. *JAMA Network Open.*

eTable 8. Coefficients from adjusted logistic regression models predicting protein inadequacy (imputed dataset, n=774).

Independent variable	Response	Odds ratio (95% CI)	P-value
Isolated protein supplement consumer	No	1.00 (reference)	-
	Yes	0.05 (0.02-0.11)	<.001
Textured soy protein consumer	No	1.00 (reference)	-
	Yes	0.29 (0.16-0.50)	<.001

Independent variable	Quartile	Value	Odds ratio (95% CI)	P-value
Unprocessed and minimally processed food	1st quartile	< 53.8 % TEI [a]	1.00 (reference)	-
	2nd quartile	66.5 % TEI	0.94 (0.51-1.73)	.85
	3rd quartile	76.8 % TEI	0.96 (0.52-1.77)	.90
	4th quartile	> 76.8 % TEI	1.46 (0.80-2.67)	.22
Ultra-processed food	1st quartile	< 4.4 % TEI	1.00 (reference)	-
	2nd quartile	13.2 % TEI	0.34 (0.18-0.65)	.001
	3rd quartile	23.2 % TEI	0.20 (0.10-0.38)	<.001
	4th quartile	> 23.2 % TEI	0.19 (0.10-0.36)	<.001
Unprocessed and minimally processed protein	1st quartile	< 44.8 % TEI	1.00 (reference)	-
	2nd quartile	61.8 % TEI	3.66 (1.87-7.34)	<.001
	3rd quartile	80.7 % TEI	4.73 (2.38-9.66)	<.001
	4th quartile	> 80.7 % TEI	12.12 (6.08-25.25)	<.001
Ultra-processed protein	1st quartile	< 5.9 % TEI	1.00 (reference)	-
	2nd quartile	23.6 % TEI	0.60 (0.32-1.12)	.11
	3rd quartile	41.7 % TEI	0.24 (0.12-0.47)	<.001
	4th quartile	> 41.7 % TEI	0.06 (0.03-0.12)	<.001

All models were adjusted for body weight, sex, age and energy intake relative to body weight. [a] TEI = total energy intake.

© 2024 Leitão AE et al. *JAMA Network Open.*

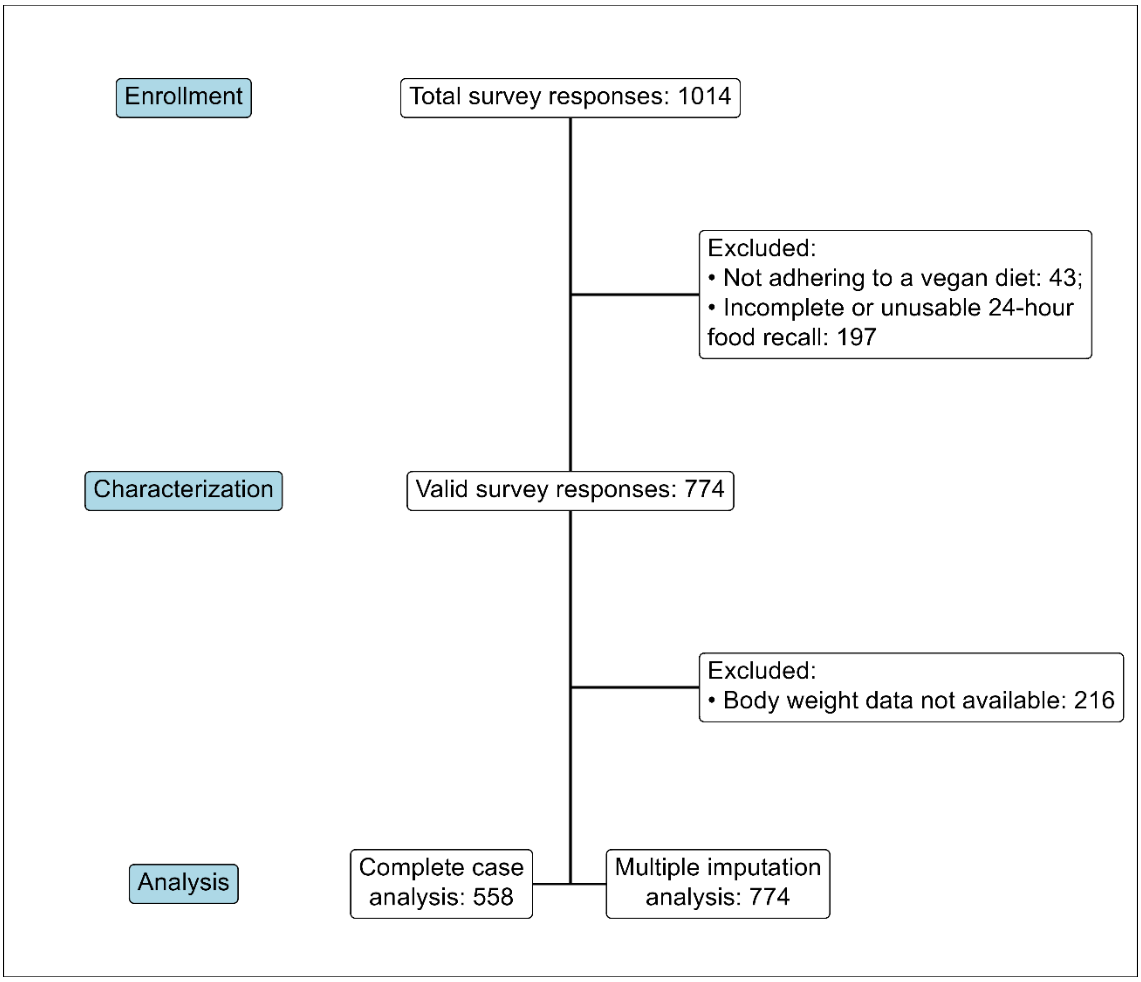

eFigure 1. Study flowchart.

© 2024 Leitão AE et al. *JAMA Network Open.*

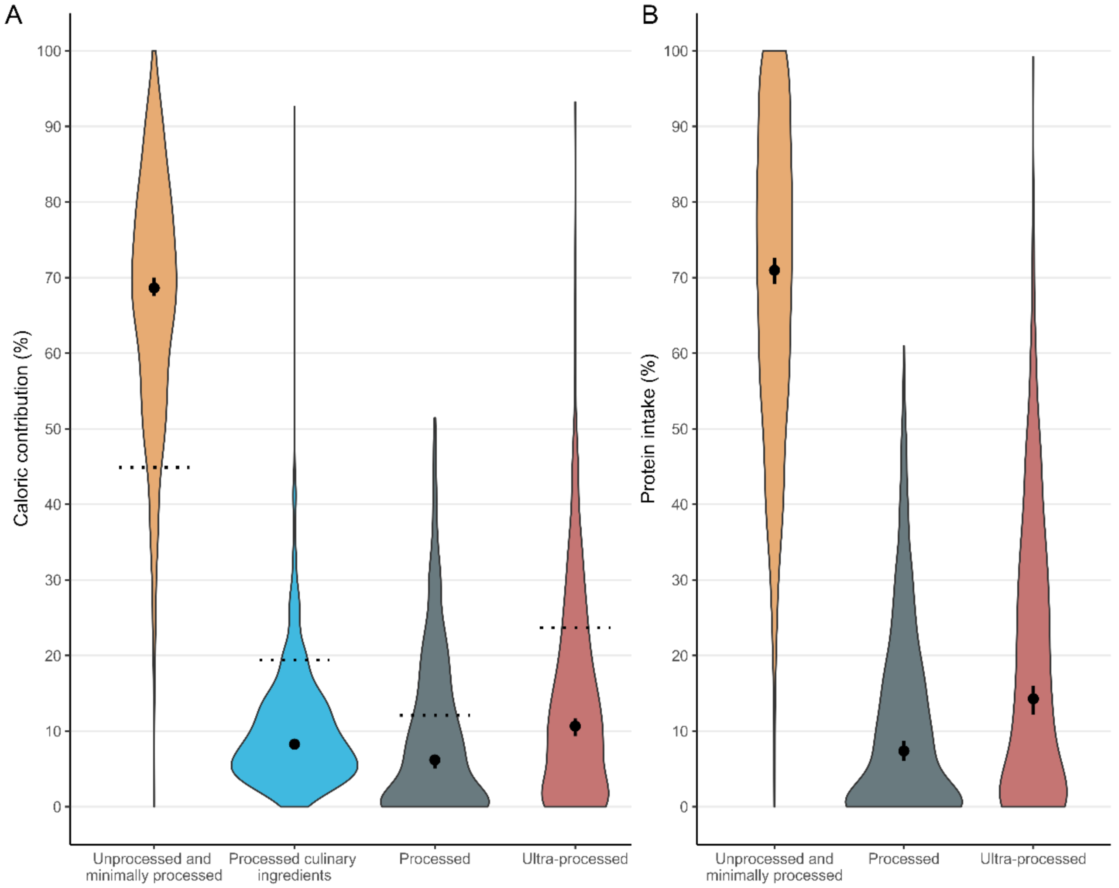

eFigure 2. Caloric and protein intake according to Nova food processing categories (considering textured soy protein as unprocessed and minimally processed).
Caption: Panel A: Violin plots showing the distribution of caloric contribution of each food processing category. Dashed line show reference values from the Brazilian population living in metropolitan areas. Panel B: protein intake contribution of each food processing category. Dots are medians accompanied by 95% confidence intervals.

© 2024 Leitão AE et al. *JAMA Network Open.*

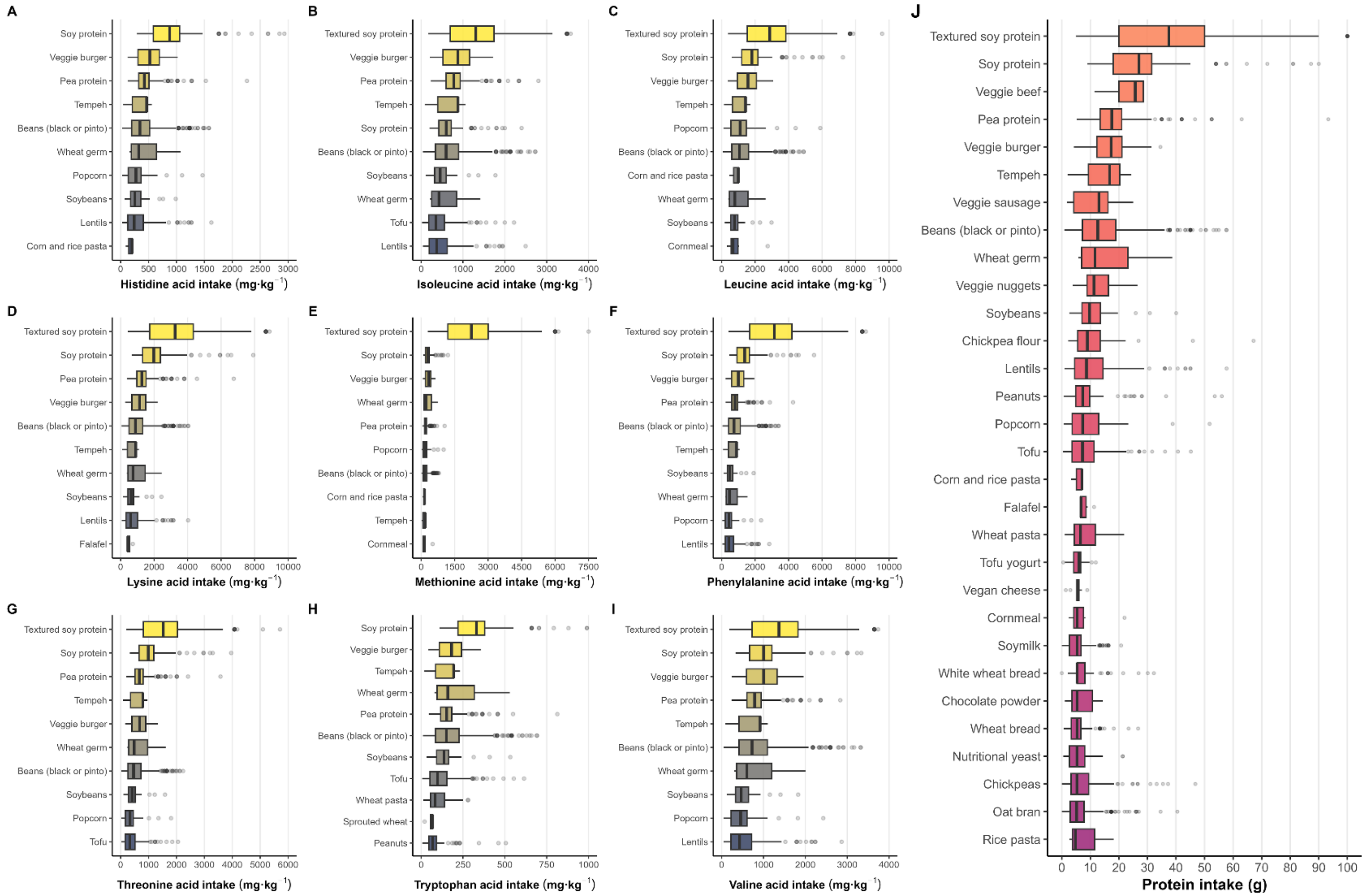

eFigure 3. Main food sources of protein and essential amino acids.

Caption: Boxplots showing protein and essential amino acid intake contribution for the top 30 and top 10 foods, respectively. Panel A: Histidine intake. Panel B: Isoleucine intake. Panel C: Leucine intake. Panel D: Lysine intake. Panel E: Methionine intake. Panel F: Phenylalanine intake. Panel G: Threonine intake. Panel H: Tryptophane intake. Panel I: Valine intake. Panel J: Protein intake.

© 2024 Leitão AE et al. *JAMA Network Open.*

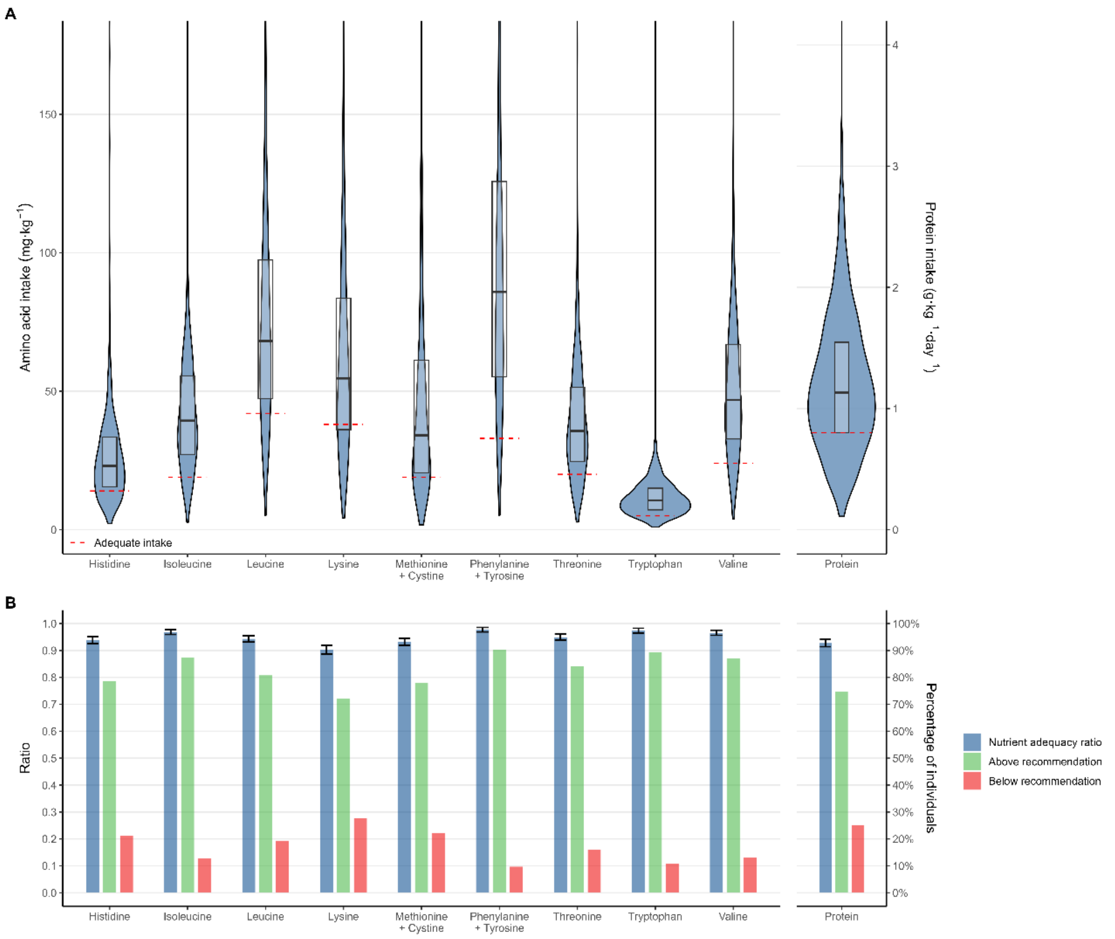

eFigure 4. Protein and essential amino acids intake, nutrient adequacy ratios and proportion of individuals meeting recommended intakes in the imputed (n=774) dataset. Caption: Panel A: Violin plots showing the distribution of essential amino acids intake relative to body mass, with the red dashed line indicating the respective recommendation and dots showing median values and 95% confidence intervals. Panel B: Nutrient adequacy ratios (presented as ratios, from 0 to 1, left axis), and proportion of individuals above or below recommendations for essential amino acids and protein (presented as percentage, 0 to 100%, right axis). Missing body weight values (n = 216) were multiply imputed, totalling n = 774.

© 2024 Leitão AE et al. *JAMA Network Open.*

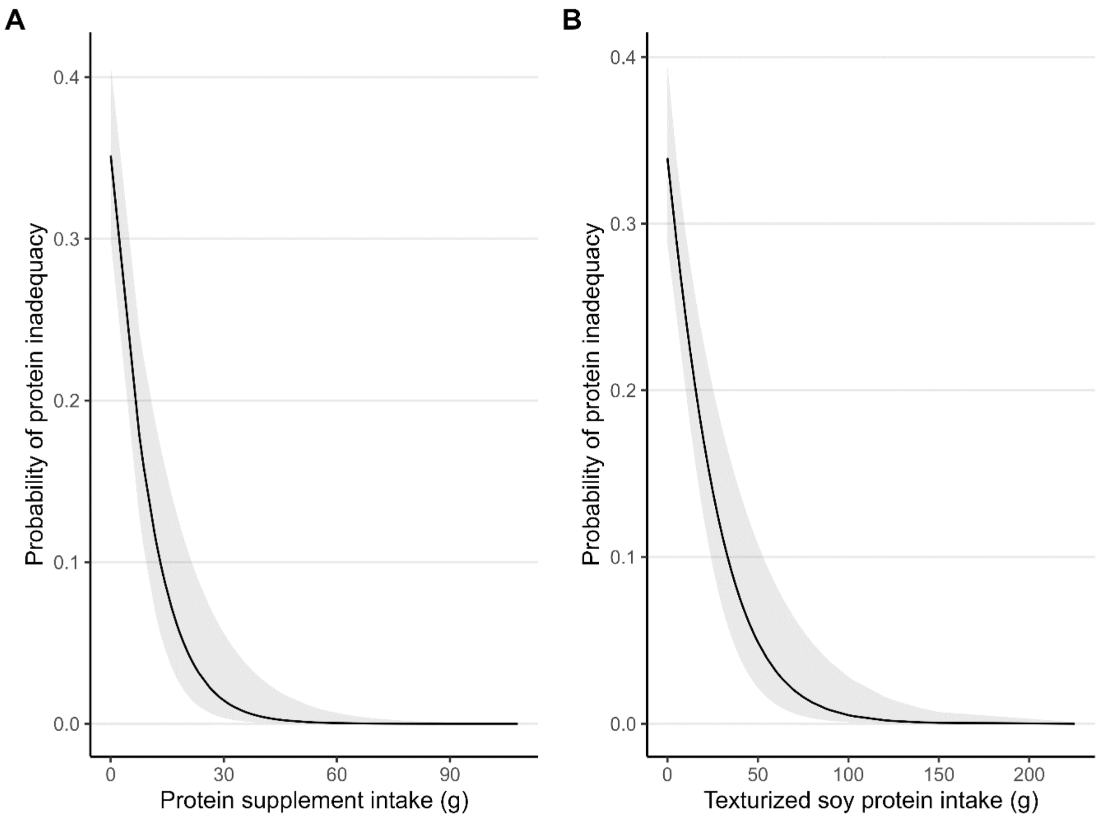

eFigure 5. Exploratory logistic regression models predicting the probability of protein inadequacy according to protein supplement intake or texturized soy protein intake as continuous variables.
Caption: Panel A: protein supplement intake; Panel B: texturized soy protein intake. Models were adjusted for body weight, sex, age and energy intake relative to body weight.

© 2024 Leitão AE et al. *JAMA Network Open.*
