# Supplementary material for: Protein and Amino Acid Adequacy and Food Consumption by Processing Level in Vegans in Brazil
Source: JAMA Netw Open. 2024 Jun 24;7(6):e2418226. doi: 10.1001/jamanetworkopen.2024.18226 (PMC11197455; doi:10.1001/jamanetworkopen.2024.18226)
Supplement: Supplement 2. — Data Sharing Statement [file jamanetwopen-e2418226-s002.pdf]

Data Sharing Statement

Leitão. Protein and Amino Acid Adequacy and Food Processing in Vegan Diets in Brazil. *JAMA Netw Open*. Published online June 24, 2024. doi:10.1001/ jamanetworkopen.2024.18226

Data

Data available: Yes
Data types: Deidentified participant data
How to access data: Deidentified participant data and statistical code may be shared upon request. Email for request: hars@usp.br.
When available: With publication

Supporting Documents

Document types: Statistical/analytic code
How to access documents: Deidentified participant data and statistical code may be shared upon request. Email for request: hars@usp.br.
When available: With publication

Additional Information

Who can access the data: Data and statistical code may be shared to researchers whose proposed use of the data has been approved.
Types of analyses: Data may be shared for the purposes of additional analyses that complement or increase the robustness of the initial analysis.
Mechanisms of data availability: Data will be made available with investigator support and after approval of a proposal has been met.
